# Aberrant MicroRNA Expression in Endometrial Carcinoma Using Formalin-Fixed Paraffin-Embedded (FFPE) Tissues

**DOI:** 10.1371/journal.pone.0081421

**Published:** 2013-12-09

**Authors:** Taek Sang Lee, Hye Won Jeon, Yong Beom Kim, Young A. Kim, Min A. Kim, Soon Beom Kang

**Affiliations:** 1 Department of Obstetrics and Gynecology, SMG–SNU Boramae Medical Center, Seoul, Korea; 2 Department of Obstetrics and Gynecology, Seoul National University College of Medicine, Seoul National University Bundang Hospital, Seongnam, Korea; 3 Department of Pathology, SMG–SNU Boramae Medical Center, Seoul Korea; 4 Department of Pathology, Seoul National University College of Medicine, Seoul, Korea; 5 Department of Obstetrics and Gynecology, Konkuk University Medical Center, Seoul, Korea; University of Nevada School of Medicine, United States of America

## Abstract

This study aimed to identify the candidate miRNAs in the carcinogenesis of endometrial carcinoma, and to explore whether FFPE material would be suitable for miRNA profiling. We identified the differences between miRNA expression profiles using human miRNA microarray in endometrioid endometrial adenocarcinomas (EECs) and normal endometria. Of those tested, miR-200a*, miR-200b*, miR-141, miR-182, and miR-205 were greatly enriched. The expressions of these five miRNAs were validated using quantitative real-time reverse transcription-PCR (qRT-PCR). We then performed qRT-PCR miR expression profiling in 30 FFPE specimens (20 EECs, 10 normal endometria) and re-confirmed the results of differential expression between cancer and normal tissue. Following this, we tested whether the specific inhibition of overexpressed miRNAs would alter chemosensitivity. In the *in vitro* cell viability assay, anti-miR200b* showed a trend toward enhanced cytotoxicity slightly in cisplatin compared to the negative control (p = 0.07). This information provided the candidate miRNAs for further confirmation of the role of miRNAs in the carcinogenesis of EECs, potentially serving as a diagnostic or therapeutic tool. FFPE specimens of endometrial tissues are suitable as a source for miRNA microarray profiling.

## Introduction

MicroRNAs (miRNAs) are small non-coding RNAs of 19–24 nucleotides that regulate gene expression post-transcriptionally by imperfect base-pairing to messenger RNAs [1]. Hundreds of miRNAs have been identified in various animal genomes. Each miRNA is believed to target as many as two hundred transcripts and approximately 30% of human genes are regulated by miRNAs [2]. The biological functions of most miRNAs are not fully understood. However, it has been suggested that miRNAs are involved in various biological processes, including cell proliferation, apoptosis, differentiation, and metabolism [3], [4]. Since the discovery in 2002 that miRNA dysregulation is linked to cancer, [5] many studies have sought to describe the relationship between miRNAs, cancer progression, and metastasis [6].

Endometrial carcinoma is the most common gynecologic malignancy in Western countries and the fourth most common cancer among women worldwide [7], [8]. Endometrioid adenocarcinoma is the main form of endometrial carcinoma, accounting for 75–80% of cases [9]. Although focusing on unopposed estrogen stimulation of the endometrium to carcinogenesis has already yielded much information, the detailed network of events leading from estrogen stimulation to tumor development has not been clarified. Some studies have shown that ovarian steroids can influence the expression of miRNAs in the endometrium and carcinogenesis of endometrial cancer is considered to be related to the progressive accumulation of multiple genetic abnormalities, which may activate oncogenes and inactivate tumor suppressor genes [10], [11]. Therefore, miRNAs may play an important role in the carcinogenesis of endometrial carcinoma.

While studies on miRNAs so far have tended to focus on fresh-frozen (FF) tissues, other types of samples, such as formalin-fixed paraffin-embedded (FFPE) samples, are being explored [12], [13]. FFPE samples have significant value as they are often the only source of tissue available from large patient cohorts with comprehensive clinical data and long-term follow-up. With this in mind, we present the results of miRNA deregulation in a set of endometrioid endometrial cancer (EEC) and normal endometrial tissues from both FF and FFPE samples using real-time quantitative PCR array.

## Materials and Methods

### Patients and tissue specimens

Fresh-frozen biopsy specimens from patients with EECs (n = 4) and normal endometrial specimens from patients who underwent a hysterectomy to treat other benign disease (n = 4) were collected. After surgical removal, the tissues were frozen immediately in liquid nitrogen and stored at −80°C. FFPE tissues were also obtained from the hysterectomy specimens of 20 endometrial cancer patients, as well as 10 normal endometrial tissues from endometrial curettage specimens. Ethical approval was obtained from the institutional review board of Seoul Metropolitan Government Seoul National University Boramae Medical Center for this study and written informed consent was obtained from all patients who provided tissues used in this study.

### RNA isolation and gene expression profiling

In this study, we performed global miRNA gene expression analyses using Agilent Human miRNA Microarray Version 3 (Agilent design IDs 021827), including 866 human and 89 human viral miRNAs. The sample preparation was performed according to the instructions and recommendations of the manufacturer. Total RNA was isolated using Trizol, as described by the manufacturer (Ambion). RNA quality was assessed by Agilent 2100 Bioanalyzer using the RNA 6000 Nano Chip (Agilent Technologies, Amstelveen, the Netherlands) and quantity was determined by ND-1000 Spectrophotometer (NanoDrop Technologies, Inc., DE, USA).

For each RNA sample, 100 ng of total RNA was used as input into the labeling reaction and the labeled miRNA was hybridized to the array for 20 hours at 55°C and 20 rpm, as recommended in the protocol (miRNA Microarray System with miRNA Complete Labeling and Hyb Kit protocol 2.1). After hybridization, the chips were washed and scanned using an Agilent DNA Microarray Scanner.

The microarray data was analyzed using SAM software version 3.08 to select the differentially expressed miRNAs. The main benefit of the SAM approach is the computation of the false discovery rate (FDR). The FDR represents the percentage of genes identified as significant by chance. A low FDR for a significant gene would indicate a greater likelihood that the gene represents a true, significant gene, rather than a falsely discovered one.

### Data analysis for microarray

Three normalization methods (total intensity, global median, and cross-correlation) were used to identify the best method for correcting systematic biases in the array data. Differential expression of miRNA gene selection was performed using the Significance Analysis of Microarrays (SAM, Stanford, California, USA) approach (Thomson et al., 2004). Hierarchical clustering was performed using Cluster 3.0 software (Stanford, California, USA) to assess expression trend.

### Real-time RT-PCR

To validate the results of miRNA array, the five most differentially expressed miRNAs were subjected to real-time RT-PCR. The cDNA was synthesized from the miRNA using a Mir-X miRNA First-strand Synthesis and SYBR qRT-PCR Kit (Takara Bio Inc). Quantitative real-time PCR with a Takara Thermal Cycler Dice (TP800)® used primers and templates mixed with the SYBR Premix. The sequence of the miRNA primer was designed based on miRBase (http://www.mirbase.org). Primer sets for individual miRNAs are listed in Table 1. These primers were pre-validated to generate single amplicons. DNA was amplified for 40 cycles of denaturation for 5 s at 95°C and annealing for 20 s at 60°C. The data generated from each PCR reaction was analyzed using the Thermal Cycler Dice Real Time System ver 2.10 B (Takara Bio Inc.) software package. We used small nucleolar RNA U6 (Ambion, Austin, Texas, USA) as an internal control and the expression of miRNAs was quantified as ΔCt values, where Ct =  cycle threshold, ΔCt =  (Ct target miRNAs − Ct u6). ΔCt was calculated using RQ manager software, version 1.2 (Applied Biosystems).

**Table 1:** PCR primers for miRNAs.

Target miRNA	Mature miRNAs Sequence	Forward primer
hsa-miR-200a^*^	CAUCUUACCGGACAGUGCUGGA	**CATCTTACCGGACAGTGCTGGA**
hsa-miR-205	UCCUUCAUUCCACCGGAGUCUG	**TCCTTCATTCCACCGGAGTCTG**
hsa-miR-141^*^	CAUCUUCCAGUACAGUGUUGGA	**CATCTTCCAGTACAGTGTTGGA**
hsa-miR-200b^*^	CAUCUUACUGGGCAGCAUUGGA	**CATCTTACTGGGCAGCATTGGA**
hsa-miR-182	UUUGGCAAUGGUAGAACUCACACU	**TTTGGCAATGGTAGAACTCACACT**

### qRT-PCR for formalin-fixed paraffin-embedded (FFPE) tissues

To generalize the results of differential expression between cancer and normal tissue, and to determine whether miR analysis of FFPE tissues of endometrial cancer is feasible, quantitative real-time PCR (qRT-PCR) miRNA expression profiling was performed in 30 archival FFPE specimens (20 cases of endometrioid adenocarcinoma described in Table 2 and 10 normal endometria obtained from office-based biopsy for benign disease).

**Table 2:** Patient characteristics of FFPE specimens diagnosed with endometrioid adenocarcinoma used to validate the miRNA microarray.

Case No.	Age	Histology	Stage	Grade	LN invasion	Recurrence
C1	61	Endometrioid	IIb	1	−	−
C2	34	Endometrioid	IIIc	2	+	−
C3	37	Endometrioid	IVb	2	+	+
C4	69	Endometrioid	IIIa	3	−	−
C5	80	Endometrioid	Ic	3	−	−
C6	73	Endometrioid	IIb	2	−	−
C7	57	Endometrioid	IIIc	2	+	−
C8	65	Endometrioid	Ic	1	−	−
C9	71	Endometrioid	Ic	1	−	−
C10	67	Endometrioid	IIIc	2	+	−
C11	56	Endometrioid	IIa	1	−	−
C12	60	Endometrioid	Ic	1	−	−
C13	60	Endometrioid	Ic	3	−	+
C14	54	Endometrioid	Ic	3	−	−
C15	54	Endometrioid	IIIc	3	+	−
C16	54	Endometrioid	IIIc	2	+	−
C17	44	Endometrioid	IIIc	2	+	−
C18	30	Endometrioid	IIIc	3	+	−
C19	59	Endometrioid	IIIc	3	+	+
C20	52	Endometrioid	Ic	1	−	−

FFPE tissues blocks were sectioned at 10 µm and evaluated by a pathologist. Regions of invasive carcinoma were confirmed and marked on each slide. Marked regions were microdissected using a new, sterile, disposable scalpel blade and the dissected tissue placed immediately into a labeled RNase-free microcentrifuge tube with a closed cap. For the 10 normal endometrial tissues from the office-based biopsy, each thin, curled-up tissue strip was placed directly into the tube and microdissection was not required.

Total RNA was extracted using Recoverall (Ambion, Austin, TX, USA), according to the manufacturer's instructions. Xylene was added to the FFPE samples briefly to remove paraffin. The tissues were then digested with protease and treated with DNase. After washing, the RNA, including the small miRNA fraction, was eluted with distilled water. The concentrations and quality of the RNA recovered were measured using the Nanodrop 1000 A spectrophotometer (Nanodrop Technologies, *Wilmington, DE, USA)*. Mean ratio of 260/280 was 1.81±0.032 (range: 1.79∼1.88) and 260/230, 1.87±0.296 (range: 1.23∼2.15).

### Cell lines and transfection of anti-miR

The human endometrial cancer cell lines, Hec1A and Ishikawa were obtained from ATCC (American Type Culture Collection, Manassas, VA, USA) and ECACC (European Collection of Cell Cultures, Wiltshire, UK), respectively. Both cell lines were prepared as follows: Hec1A cells were grown at 37°C in 5% CO2 in RPMI 1640 supplemented with 15% fetal bovine serum, penicillin (100 units/ml), and streptomycin (100 Ag/ml); and Ishikawa cells were maintained in Dulbecco's Modified Eagle's Medium/Ham's Nutrient Mixture F-12 (DMEM/F12).

The miR-inhibitor solutions (0, 25, 50 nM) (Ambion, Austin, TX, USA) were stocked briefly in a 50 µl RNase-free, pH 7.4-buffered solution. 2 µl of G-fectin reagent (Genolution Pharmaceuticals) were then added and the solutions incubated for 10 minutes at room temperature. The cells were split and 450 µl of media with cells were added to 96-well plates with 30–50% cell confluency for optimum transfection efficacy. After 10 minutes, lipoplex was added to each intended plate. Assays was performed 24∼48 hours after transfection. To further assess whether the miR inhibitors affected the cytotoxic effect of chemotherapeutic agents, we treated the transfected cells with cisplatin and paclitaxel.

Cell viability was monitored using the 2-(4,5-dimethyltriazol-2yl)-2,5-diphenyltetrazolium bromide (MTT, Sigma, St. Louis, MO, USA) colormetric assay; 5 mg/ml of MTT solution were added to each well. After four hours of incubation at 37°C, the cell supernatants were not discarded. MTT crystals were dissolved with DMSO and the absorbance measured at 570 nm. All experiments were conducted with 24 wells per experiment and repeated at least three times.

### Statistical analysis

Statistical analysis was performed using Statistical Package for Social Sciences (SPSS) software 18.0 (SPSS, Inc., an IBM Company, Chicago, Illinois, USA). The Mann–Whitney test was used to analyze the differences in miRNA expression between the tumor and non-tumor tissues. A p-value calculated by one-tailed test of less than 0.05 was considered statistically significant.

## Results

### Distinct miRNA signatures in microarray

**Figure 1:**
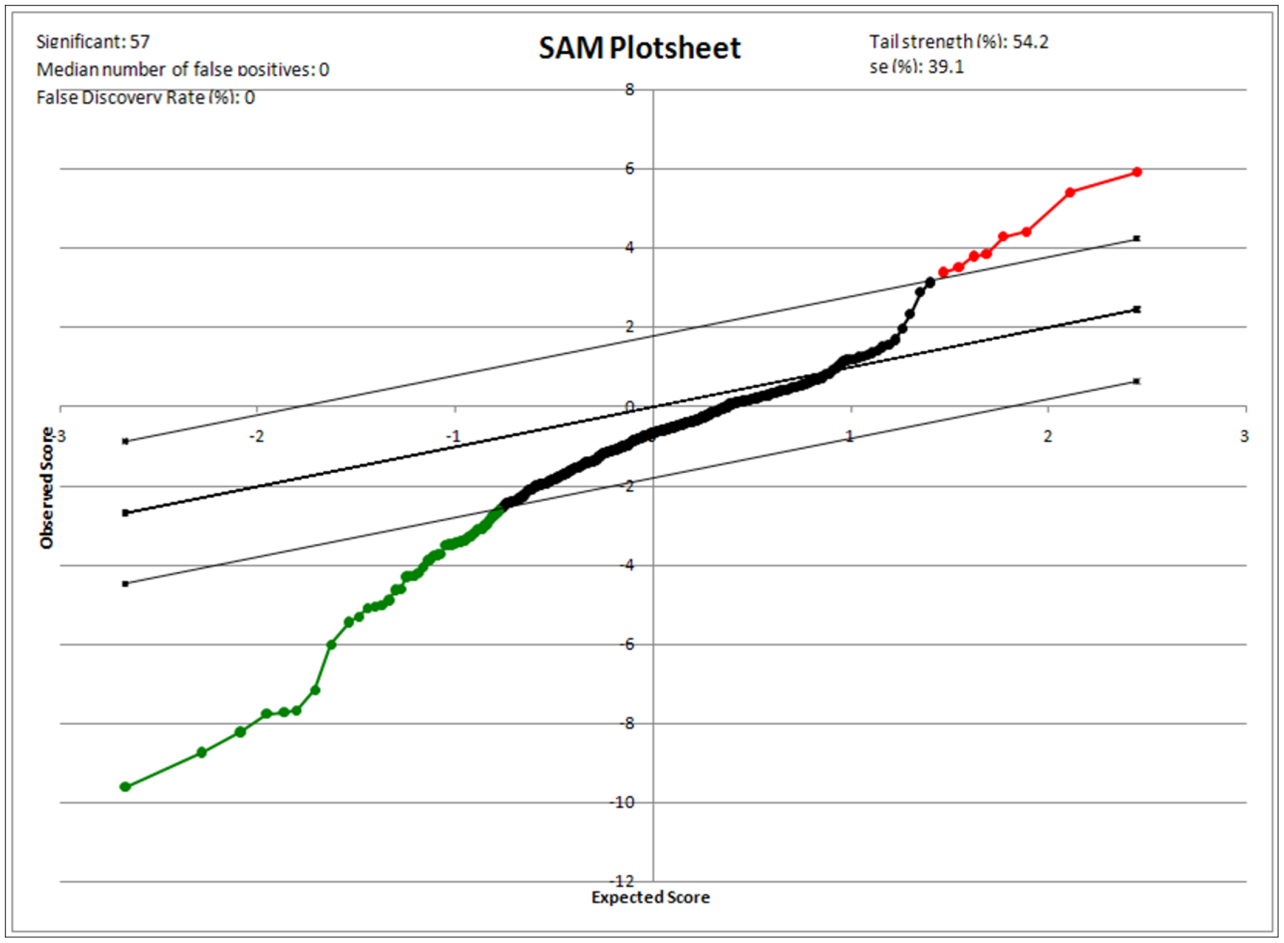
Significance analysis of microarrays (SAM) plotsheet showing the relative expression of miRNA genes from paired-class t-test. foot note: Red dots indicate highly expressed genes and green dots indicate lower expressed genes with 1.5-fold change.

**Figure 2:**
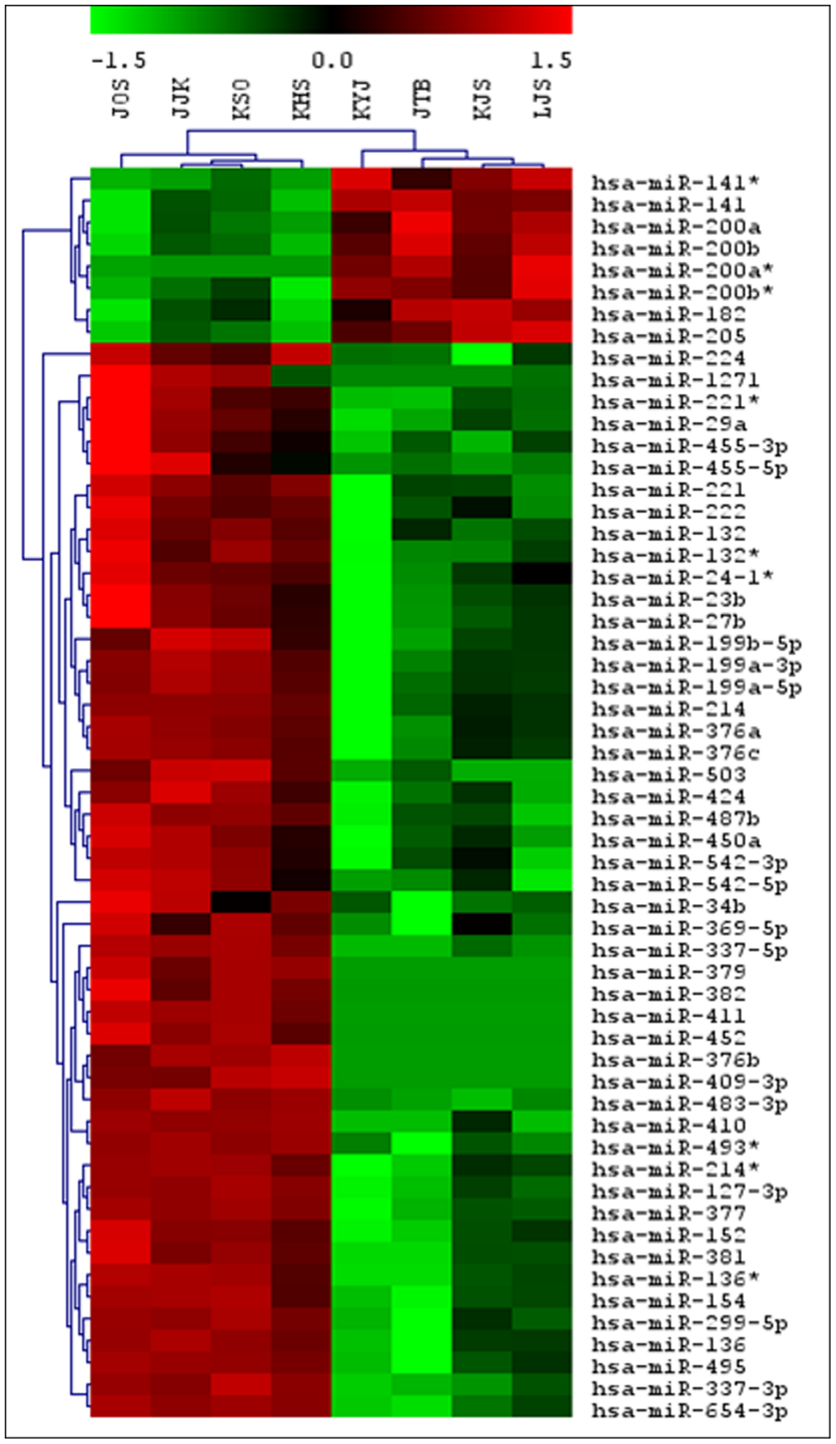
Unsupervised hierarchical clustering of four endometrioid adenocarcinoma and four normal endometrial tissues. foot note: Samples were clustered according to the expression profile of 57 miRNAs differentially expressed between tumor (right four) and non-tumor (left four) samples. The level of miRNA expression is color-coded. *Red*, higher miRNA expression; *green*, lower miRNA expression; *black*, no difference.

We analyzed the miRNA expression profiles of four endometrioid adenocarcinoma and four normal endometrial tissues using a two-color system. When the selective conditions were a FDR of less than 0.05 and the fold change was 2.87, there were eight human miRNA genes up-regulated and 49 down-regulated by SAM analysis (Fig. 1). The hierarchical clustering was performed after SAM analysis (Fig. 2). The up-regulated miRNA was miR-200a* miR-205, miR-141*, miR-200b*, miR-141, miR-200b, miR-200a, miR182. Among these miRNAs, five were up-regulated in tumor samples where the fold change was more than 5-fold (miR-200a*, miR-205, miR-141*, miR-200b*, miR182).

### Validation of the microarray results with cell lines, FF, and FFPE tissues

Five miRNA genes (miR-200a*, miR-205, miR-141*, miR-200b*, miR182) were selected because of their notably high differential expression in tumor tissues in the microarray data. The expression of the five miRNA genes was determined using real-time PCR analysis.

**Figure 3:**
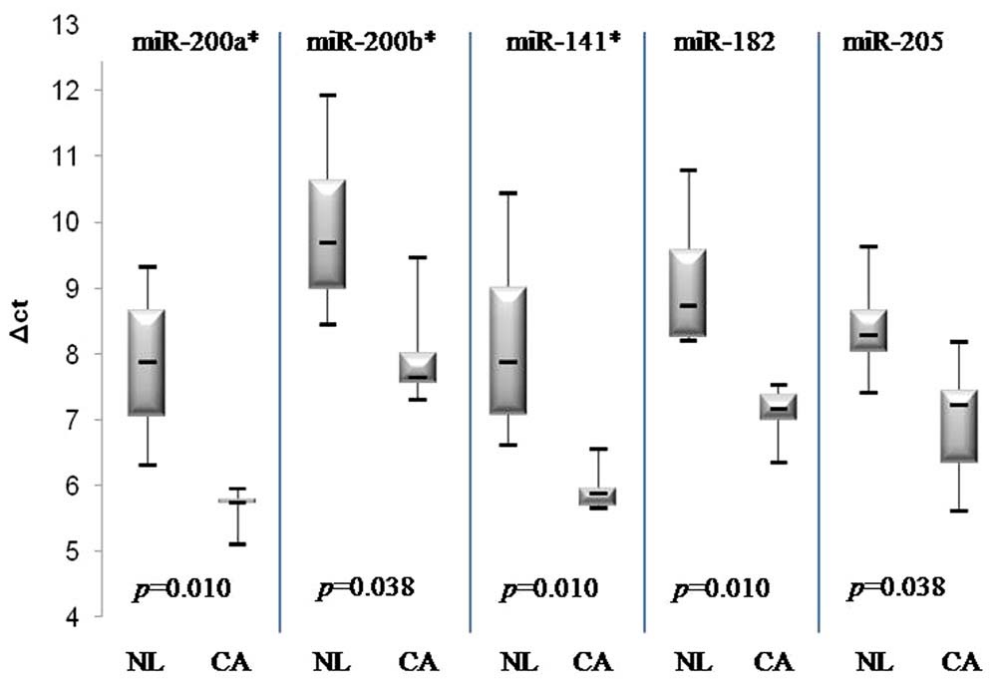
Up-regulated expression of five miRNAs in fresh-frozen endometrial cancer tissues and cells. foot note: NL, Normal; CA, Cancer. *p*-values were obtained using the Mann–Whitney test.

**Figure 4:**
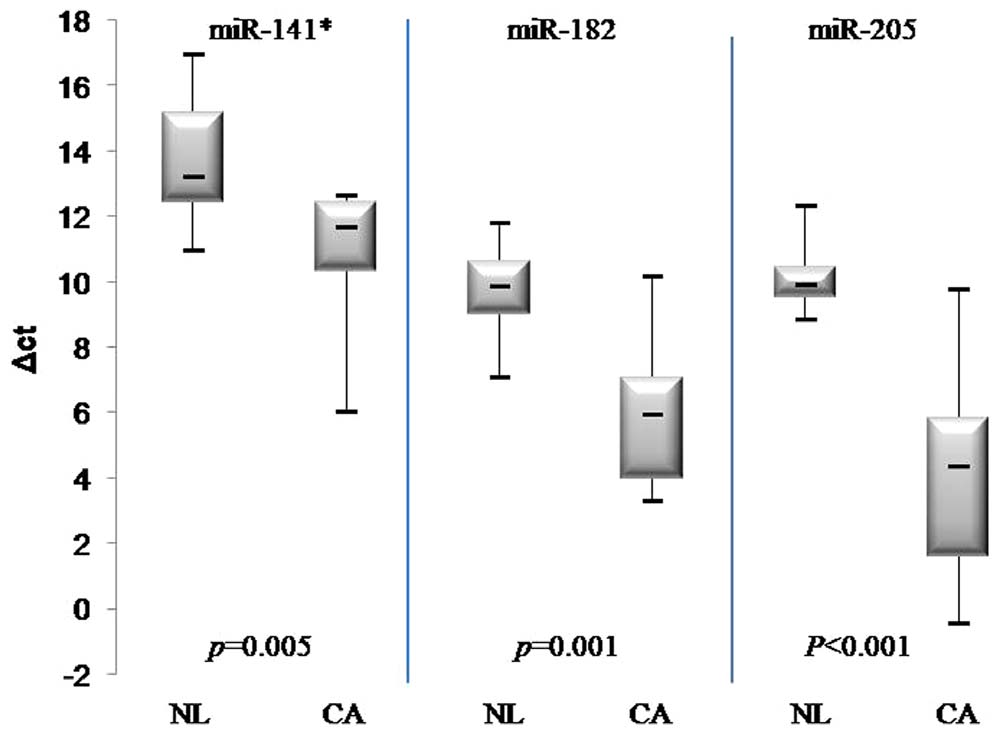
Up-regulated expression of three miRNAs in FFPE-derived tissues including 20 endometrial cancer tissues and 10 non-neoplastic tissues. foot note: NL, Normal; CA, Cancer. *p*-values were obtained using the Mann–Whitney test.

In line with the microarray results, the expression levels of the five miRNAs were much higher in endometrial cancer tissues and endometrial cancer cell lines (Hec1A, Ishikawa) than in non-tumor tissues. (Fig. 3) To generalize the above results and examine whether the miRNA sequencing could be applied to FFPE-derived samples, we investigated the expressions of three miRNAs (miR-141*, miR-205, miR182) using 20 microdissected FFPE endometrial cancer samples and 10 non-tumor endometrial FFPE tissues. The miR-200 family and miR-141 are known to have similar seed sequences, meaning that it is highly probable that they share target genes. Therefore, we selected only miR-141 (instead of miR-200a* or 200b*) for the validation study with FFPE-derived tissues. The altered expressions of all three miRNAs between tumor and benign samples were highly consistent with the data from the fresh-frozen tissues (Fig. 4).

### Relationship between miRNA expressions and various clinicopathologic factors

In total, 20 microdissected FFPE samples of endometrioid carcinoma were used to investigate the relationships between overexpressed miRNAs and a variety of prognostic factors including age, tumor grade, and lymph node metastasis. As shown in Table 3, we found no statistically significant correlation between ΔCt value of miRNAs and the prognostic factors.

**Table 3:** Relationship between miRNA expressions and various clinicopathologic prognostic factors.

	ΔCt	ΔmiR-205	p-value	ΔmiR-141*	p-value	ΔmiR-182	p-value
Age	≥60	3.74	0.603	11.41	0.883	5.86	1.000
	<60	4.26		10.67		5.97	
Recurrence	Yes	2.25	0.305	10.10	0.600	5.20	0.800
	No	4.28		10.98		6.02	
LN invasion	Yes	4.93	0.370	10.07	0.610	5.26	0.476
	No	3.19		11.44		6.39	
Grade	1	2.40	0.130	10.67	0.667	4.39	0.183
	2,3	4.65		10.99		6.60	

ΔCt =  Ct (miR) – Ct(u6); LN, lymph node.

### Cell viability assay

**Figure 5:**
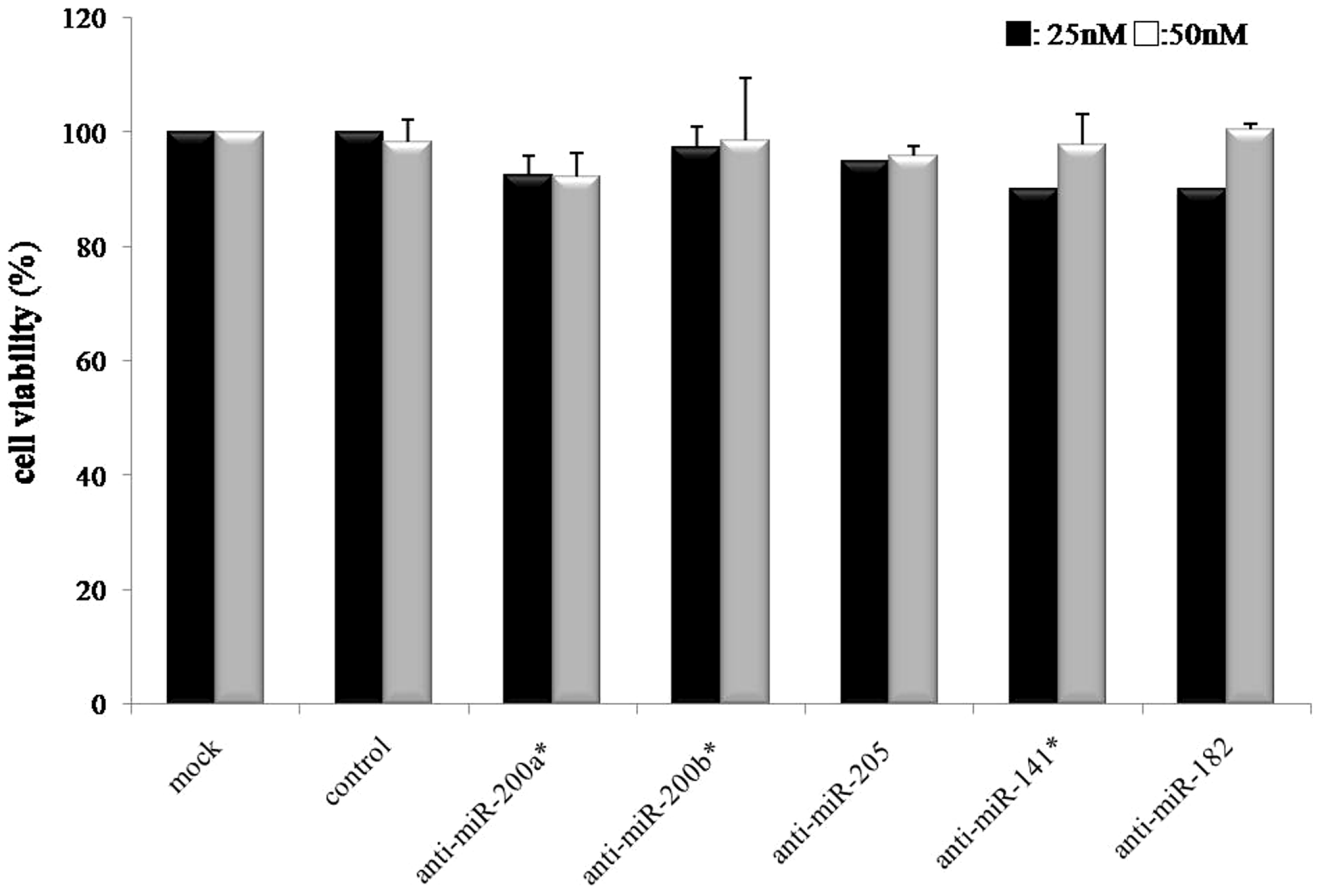
Cell viability assay with Hec1A cell line for the miR inhibitors at 25, 50 Values are mean of three independent experiment ±S.E. (p = NS).

To investigate the cellular mechanism for the hypothesis that high miRNA expression is associated with tumor cell progression, we transfected the three miR inhibitors (miR-141*, miR-205, miR-182) into the endometrial cancer cells (Hec1A) to down-regulate miRNA activity. After 72 hours of incubation in the 25 nM and 50 nM miR-inhibitor concentrations, cell proliferation was not inhibited by any miR-inhibitor in the MTT assay. (Fig. 5).

### Effect of specific inhibition with anti-miRNAs on chemosensitivity

**Figure 6:**
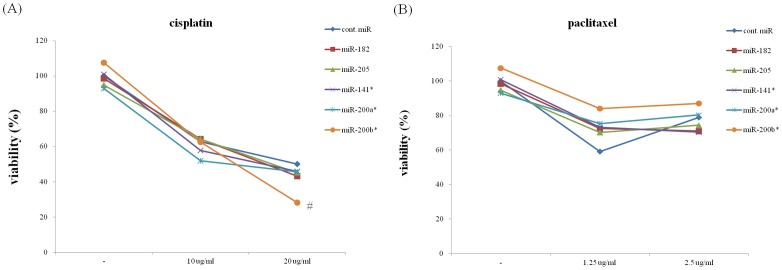
Effects of specific anti-miRs for the five miRNAss on the IC50 of cisplatin (A) and paclitaxel (B) in Hec1A cells. foot note: # anti-miR200b* slightly decreased total cell count with marginal significance in cisplatin treatment. *p* = 0.07 vs. negative control (*n* = 3 experiments), the other four did not show significant difference compared with the negative control inhibitor.

To address the hypothesis that miR overexpression is involved in chemosensitivity, we tested whether the specific inhibition of overexpressed miRNAs alters the sensitivity of cells to cisplatin or paclitaxel. We transfected with the five anti-miRNAs (anti-miR-141*, anti-miR-205, anti-miR-182, anti-miR-200a*, anti-miR-200b*) into the Hec1A cells and treated them with IC50 of cisplatin or paclitaxel for 48 hours. As shown in Fig. 6, anti-miR200b* showed a trend toward enhanced cytotoxicity in cisplatin compared to the negative control in three repeated experiments (p = 0.07). However, it did not show any effect on the cytotoxicity of paclitaxel.

## Discussion

In this study, we identified several altered miRNAs expressions in endometrial cancer tissues and normal endometrial tissues.

Our data shows some similarity with previously published reports. The up-regulation of the miR-200 family (miR-141, 200a, 200b) is observed consistently, despite the use of different patient populations and different tissue types (fresh vs. formalin-fixed) in EEC relative to normal controls [14]–[18].

This miR-200 family is composed of five miRNAs localized on two genomic clusters: miR-200c and miR-141 on chromosome 12 and miR-200a, miR-200b and miR-429 on chromosome 1. Members of this family are known to be involved in the epithelial to mesenchymal transition (EMT) that occurs during tumor invasion and metastasis in uterine and other tissues [19]. Coordinately regulated with the miR-200 family, miR-205 is one of the consistently over-expressed miRNAs in EEC. Although our data did not include the data associated with the correlation between overexpressed miRNAs and clinical prognostic factors due to the limited case number and recurrent cases, the aberrant expression of miR-205 was reported to be correlated with advanced-stage disease and poor patient survival rates in endometrial cancer [15], [20].

Up-regulated miR-182 in endometrial cancer is reported to be associated with the repressing FOXO1 gene, which is known to be down-regulated in endometrial cancer compared to normal endometrial [21], [22]. The ability of miR-182 to promote FOXO1 repression may adopt a key role in endometrial tumorigenesis by bypassing the cell cycle and cell death control.

It is standard practice for pathology departments to store almost all human samples in FFPE blocks; these can be linked with clinical databases, including disease diagnoses and patient follow-up information. The use of FFPE samples is always challenging but is extremely beneficial, as it is almost the only way to make large-scale and clinical trial/cohort-based studies possible. In recent years, FFPE specimens have been used for real-time PCR-based quantitative miRNA expression studies [23]–[25]. Since these samples are assumed to have insufficient quality and integrity of RNA, they have not been used for whole-genome, small, non-coding RNA sequencing analysis to characterize miRNA expression. In our study, miRNAs could be detected in both frozen and FFPE samples. We also found that miRNAs are relatively stable in FFPE samples even in the curettage specimens collected on an outpatient-basis. One hypothesis is that they are too small to be degraded [25], [26]; however, this hypothesis has not been supported by any reported data. As we now know, active, mature miRNAs are processed and function via binding to Argonaute family proteins [27], [28]. These protein–miRNA complexes may protect the functional population of miRNA from degradation, especially during the process of formalin fixation and storage in paraffin. The anticipated benefits of using FFPE in miRNA work on endometrial cancer are: first, it is almost the only way to make large-scale and clinical trial/cohort-based studies possible; second, by using microdissection of FFPE tissues we can exclude the possibility of contamination of normal endometrial tissues, which can happen during the intraoperative collection of endometrial tumor tissues; and third, the stored endometrial tissues can be obtained easily via office-based biopsy. The future miRNA work with office based endometrial tissues may provide a basis to actualize preoperative prediction of prognosis or response for treatment.

The miR-200 family is expressed more highly in endometrial cancers than in normal endometrial tissues, and its overexpression is known to be correlated with cisplatin resistance [29]–[31]. Although we were unable to document whether the highly expressed miRNAs have an impact on cellular proliferation or apoptosis by using endometrial cancer cell lines, we identified that treatment with anti-miR200b* showed a trend toward enhanced cytotoxicity of cisplatin although we did not get statistical significance. Although not consistent, the association between the aberrant expression of miR200b and the change of response to cisplatin has been documented by other studies and we obtained similar results to those in previous reports [32], [33].

The major limitation of this study is the lack of prediction and identification of an exact target for the miRNAs validated in this study. Future research is needed to resolve these problems. If the target genes of these miRNAs are identified, the role of differential expression of the miRNAs in the carcinogenesis of endometrioid adenocarcinoma will be clarified. Another limitation is that we were unable to document the correlation between aberrant expression and the prognostic factor because of the small sample size and limited number of recurrence cases.

In summary, this study focuses on investigating the relative expression of mature miRNA genes in endometrioid adenocarcinoma and identifying a specific miRNA expression profile. We also tested whether FFPE material is suitable for miRNA profiling and obtained satisfactory results. Despite the uncertainty regarding the functional effects of these miRNAs, the miRNA expression profile may provide a basis for further study of the miRNA function in endometrioid adenocarcinoma, and be used as a prognostic marker for the aggressiveness of human cancer.

## References

[1] Lagos-Quintana M, Rauhut R, Lendeckel W, Tuschl T (2001) Identification of novel genes coding for small expressed RNAs. Science 294: 853–858. 11679670 10.1126/science.1064921

[2] Rajewsky N, Socci ND (2004) Computational identification of microRNA targets. Dev Biol 267: 529–535. 15013811 10.1016/j.ydbio.2003.12.003

[3] Chen X (2004) A microRNA as a translational repressor of APETALA2 in Arabidopsis flower development. Science 303: 2022–2025. 12893888 10.1126/science.1088060 PMC5127708

[4] Poy MN, Eliasson L, Krutzfeldt J, Kuwajima S, Ma X, et al (2004) A pancreatic islet-specific microRNA regulates insulin secretion. Nature 432: 226–230. 15538371 10.1038/nature03076

[5] Garzon R, Fabbri M, Cimmino A, Calin GA, Croce CM (2006) MicroRNA expression and function in cancer. Trends Mol Med 12: 580–587. 17071139 10.1016/j.molmed.2006.10.006

[6] Zhang B, Pan X, Cobb GP, Anderson TA (2007) microRNAs as oncogenes and tumor suppressors. Dev Biol 302: 1–12. 16989803 10.1016/j.ydbio.2006.08.028

[7] Jemal A, Ward E, Thun MJ (2007) Recent trends in breast cancer incidence rates by age and tumor characteristics among U.S. women. Breast Cancer Res 9: R28. 17477859 10.1186/bcr1672 PMC1929089

[8] Kang S, Lee JM, Lee JK, Kim JW, Cho CH, et al (2012) How low is low enough? Evaluation of various risk-assessment models for lymph node metastasis in endometrial cancer: a Korean multicenter study. J Gynecol Oncol 23: 251–256. 23094128 10.3802/jgo.2012.23.4.251 PMC3469860

[9] Pecorelli S, Pasinetti B, Angioli R, Favalli G, Odicino F (2005) Systemic therapy for gynecological neoplasms: ovary, cervix, and endometrium. Cancer Chemother Biol Response Modif 22: 515–544. 16110627

[10] Pan Q, Luo X, Toloubeydokhti T, Chegini N (2007) The expression profile of micro-RNA in endometrium and endometriosis and the influence of ovarian steroids on their expression. Mol Hum Reprod 13: 797–806. 17766684 10.1093/molehr/gam063

[11] Chen X, Yan Q, Li S, Zhou L, Yang H, et al (2012) Expression of the tumor suppressor miR-206 is associated with cellular proliferative inhibition and impairs invasion in ERalpha-positive endometrioid adenocarcinoma. Cancer Lett 314: 41–53. 21983130 10.1016/j.canlet.2011.09.014

[12] Hall JS, Taylor J, Valentine HR, Irlam JJ, Eustace A, et al (2012) Enhanced stability of microRNA expression facilitates classification of FFPE tumour samples exhibiting near total mRNA degradation. Br J Cancer 107: 684–694. 22805332 10.1038/bjc.2012.294 PMC3419950

[13] Yu J, Ohuchida K, Mizumoto K, Fujita H, Nakata K, et al (2012) MicroRNA miR-17-5p is overexpressed in pancreatic cancer, associated with a poor prognosis, and involved in cancer cell proliferation and invasion. Cancer Biol Ther 10: 748–757. 10.4161/cbt.10.8.13083 20703102

[14] Boren T, Xiong Y, Hakam A, Wenham R, Apte S, et al (2008) MicroRNAs and their target messenger RNAs associated with endometrial carcinogenesis. Gynecol Oncol 110: 206–215. 18499237 10.1016/j.ygyno.2008.03.023

[15] Chung TK, Cheung TH, Huen NY, Wong KW, Lo KW, et al (2009) Dysregulated microRNAs and their predicted targets associated with endometrioid endometrial adenocarcinoma in Hong Kong women. Int J Cancer 124: 1358–1365. 19065659 10.1002/ijc.24071 PMC6953413

[16] Wu W, Lin Z, Zhuang Z, Liang X (2009) Expression profile of mammalian microRNAs in endometrioid adenocarcinoma. Eur J Cancer Prev 18: 50–55. 19077565 10.1097/CEJ.0b013e328305a07a

[17] Ratner ES, Tuck D, Richter C, Nallur S, Patel RM, et al (2010) MicroRNA signatures differentiate uterine cancer tumor subtypes. Gynecol Oncol 118: 251–257. 20542546 10.1016/j.ygyno.2010.05.010 PMC2918705

[18] Cohn DE, Fabbri M, Valeri N, Alder H, Ivanov I, et al. (2010) Comprehensive miRNA profiling of surgically staged endometrial cancer. Am J Obstet Gynecol 202: : 656 e1–8. 10.1016/j.ajog.2010.02.051 PMC4278076 20400061

[19] Wright JA, Richer JK, Goodall GJ (2010) microRNAs and EMT in mammary cells and breast cancer. J Mammary Gland Biol Neoplasia 15: 213–223. 20499142 10.1007/s10911-010-9183-z

[20] Karaayvaz M, Zhang C, Liang S, Shroyer KR, Ju J (2012) Prognostic significance of miR-205 in endometrial cancer. PLoS One 7: e35158. 22514717 10.1371/journal.pone.0035158 PMC3325973

[21] Takano M, Lu Z, Goto T, Fusi L, Higham J, et al (2007) Transcriptional cross talk between the forkhead transcription factor forkhead box O1A and the progesterone receptor coordinates cell cycle regulation and differentiation in human endometrial stromal cells. Mol Endocrinol 21: 2334–2349. 17609436 10.1210/me.2007-0058

[22] Majid S, Dar AA, Saini S, Yamamura S, Hirata H, et al (2010) MicroRNA-205-directed transcriptional activation of tumor suppressor genes in prostate cancer. Cancer 116: 5637–5649. 20737563 10.1002/cncr.25488 PMC3940365

[23] Xi Y, Nakajima G, Gavin E, Morris CG, Kudo K, et al (2007) Systematic analysis of microRNA expression of RNA extracted from fresh frozen and formalin-fixed paraffin-embedded samples. RNA 13: 1668–1674. 17698639 10.1261/rna.642907 PMC1986820

[24] Zhang X, Chen J, Radcliffe T, Lebrun DP, Tron VA, et al (2008) An array-based analysis of microRNA expression comparing matched frozen and formalin-fixed paraffin-embedded human tissue samples. J Mol Diagn 10: 513–519. 18832457 10.2353/jmoldx.2008.080077 PMC2570634

[25] Hui AB, Shi W, Boutros PC, Miller N, Pintilie M, et al (2009) Robust global micro-RNA profiling with formalin-fixed paraffin-embedded breast cancer tissues. Lab Invest 89: 597–606. 19290006 10.1038/labinvest.2009.12

[26] Lu J, Getz G, Miska EA, Alvarez-Saavedra E, Lamb J, et al (2005) MicroRNA expression profiles classify human cancers. Nature 435: 834–838. 15944708 10.1038/nature03702

[27] Mourelatos Z, Dostie J, Paushkin S, Sharma A, Charroux B, et al (2002) miRNPs: a novel class of ribonucleoproteins containing numerous microRNAs. Genes Dev 16: 720–728. 11914277 10.1101/gad.974702 PMC155365

[28] Hutvagner G, Simard MJ (2008) Argonaute proteins: key players in RNA silencing. Nat Rev Mol Cell Biol 9: 22–32. 18073770 10.1038/nrm2321

[29] Lee JW, Park YA, Choi JJ, Lee YY, Kim CJ, et al (2011) The expression of the miRNA-200 family in endometrial endometrioid carcinoma. Gynecol Oncol 120: 56–62. 21035172 10.1016/j.ygyno.2010.09.022

[30] Hamano R, Miyata H, Yamasaki M, Kurokawa Y, Hara J, et al (2011) Overexpression of miR-200c induces chemoresistance in esophageal cancers mediated through activation of the Akt signaling pathway. Clin Cancer Res 17: 3029–3038. 21248297 10.1158/1078-0432.CCR-10-2532

[31] Wu Y, Xiao Y, Ding X, Zhuo Y, Ren P, et al (2011) A miR-200b/200c/429-binding site polymorphism in the 3′ untranslated region of the AP-2alpha gene is associated with cisplatin resistance. PLoS One 6: e29043. 22194984 10.1371/journal.pone.0029043 PMC3237583

[32] Feng B, Wang R, Chen LB (2012) Review of miR-200b and cancer chemosensitivity. Biomed Pharmacother 66: 397–402. 22795796 10.1016/j.biopha.2012.06.002

[33] Pogribny IP, Filkowski JN, Tryndyak VP, Golubov A, Shpyleva SI, et al (2010) Alterations of microRNAs and their targets are associated with acquired resistance of MCF-7 breast cancer cells to cisplatin. Int J Cancer 127: 1785–1794. 20099276 10.1002/ijc.25191

